# Prefrontal activation related to spontaneous creativity with rock music improvisation: A functional near-infrared spectroscopy study

**DOI:** 10.1038/s41598-019-52348-6

**Published:** 2019-11-05

**Authors:** Atsumichi Tachibana, J. Adam Noah, Yumie Ono, Daisuke Taguchi, Shuichi Ueda

**Affiliations:** 1 Department of Histology and Neurobiology, Dokkyo Medical University School of Medicine, Mibu, Japan; 2 Department of Psychiatry, Yale School of Medicine, New Haven, USA; 3 Department of Electronics and Bioinformatics, Meiji University, Tokyo, Japan; 4 Department of Judo Therapy, Faculty of Medical Technology, Teikyo University, Tokyo, Japan

**Keywords:** Cognitive control, Neural circuits

## Abstract

Understanding how the brain modulates improvisation has been the focus of numerous studies in recent years. Models have suggested regulation of activity between default mode and executive control networks play a role in improvisational execution. Several studies comparing formulaic to improvised sequences support this framework and document increases in activity in medial frontal lobe with decreased activity in the dorsolateral prefrontal cortex (DLPFC). These patterns can be influenced through training and neural responses may differ between in beginner and expert musicians. Our goal was to test the generalizability of this framework and determine similarity in neural activity in the prefrontal cortex during improvisation. Twenty guitarists performed improvised and formulaic sequences in a blues rock format while brain activity was recorded using functional near-infrared spectroscopy. Results indicate similar modulation in DLPFC as seen previously. Specific decreases of activity from left DLPFC in the end compared to beginning or middle of improvised sequences were also found. Despite the range of skills of participants, we also found significant correlation between subjective feelings of improvisational performance and modulation in left DLPFC. Processing of subjective feelings regardless of skill may contribute to neural modulation and may be a factor in understanding neural activity during improvisation.

## Introduction

Creativity is widely used in daily life and has been suggested to be associated with numerous brain functions, including concepts of control, memory, learning, perception and thinking^1–4^. Several studies examining aspects of improvisation and creativity related to cognition have suggested roles for multiple neural networks including executive function and resting state networks in this process. However, the interaction between these areas contribute to the creation of domain-general improvisational behavior has not been fully elucidated in detail.

A framework regarding how individuals learn to improvise and is rooted in balancing a number of ongoing neural processes has been proposed by Pressing (1998)^4,5^. Pressing suggests that improvising requires the simultaneous execution of several processes in real-time, including sensory and perceptual encoding, motor control, performance monitoring, and memory retrieval, among others. Regulation of these processes likely involves modulation of multiple cortical networks including the executive function and resting state networks^1^. Pressing also suggests that practice has the ability to automate numerous informational streams which can free attentional resources so an individual can better balance higher order processes involved in creativity and improvisation.

Support for multiple aspects of Pressings framework argue on one hand that the ability to improvise in a musical domain is an acquired skill that requires substantial training to achieve such expertise^1,6^. In contrast, it has also been suggested that that extensive practice is “necessary but not sufficient” for high-level performance associated with expertise and the ability to improvise^7,8^. Additionally, general cognitive ability or intelligence has been suggested to play a role in developing expertise in both music^9^ and interactive games such as chess^10^. While there is ample evidence for the role of intelligence^9,10^ and training^1^ in the ability to improvise musically, this does not mean that only highly practiced or highly intelligent individuals can perform improvised behaviors. Others argue that that music creativity is a skill that is potentially present in all people, and at a basic level, even beginner musicians and children have this innate ability^11,12^. One goal in the present study is to determine neural responses in musicians performing improvisation compared to more formulaic or memorized sequences that have backgrounds ranging from beginner to professional.

The last decade of neural imaging and behavioral studies have provided an understanding of not only the networks involved in the generation of improvised behavior, but also some detail on how modulation of activity within executive control centers of the prefrontal cortex (PFC) and default mode networks contribute to the specifics of the production of creative behaviors. It has been argued that the PFC is associated with a number of critical cognitive functions including creative thinking^1,13–21^ and executive function^3,22^. This role of the PFC in creative behaviors is supported in studies that show damage to the PFC impairs creativity^23^. de Souza *et al*. suggested that poor creativity is correlated with prefrontal hypoperfusion, particularly in the frontal pole, and that the integrity of the cytoarchitecture of the PFC (specifically the frontopolar area) is strongly associated with creative thought^23^.

In the domain of musical creativity, Limb and Braun attempted to describe the neural components that are involved in jazz piano improvisation^13^. Using fMRI to study trained musicians, they suggested that generation of specific patterns of distributed activation and deactivation in the prefrontal cortex may provide a cognitive context that enables the emergence of spontaneous creative behavior. They suggested that decreased dorsolateral prefrontal cortex (DLPFC) activity was associated with inhibition of conscious monitoring processes. This inhibition was further associated with increases in activity present within the frontal aspects of the default mode network which were suggested to influence internally directed actions. The authors argued this pattern reflects a combination of psychological processes required for spontaneous improvisation and may be related to modification of central processes that mediate self-monitoring and volitional control, which could be related to initiation of the creative process. Another similar study exploring musical improvisation in rap vocalists found similar results to Limb and Braun with decreased neural activity present in the dorsolateral prefrontal cortex (DLPFC) and increases in activity in the medial prefrontal cortex when contrasting improvised to formulaic or memorized rapping sequences^24^. While these similar patterns in the PFC have been repeated for both individual vocal and piano performance domains, other studies have found potentially contrasting results. One example is shown in a report where increased activation of the DLPFC was found during collaborative piano improvisation^25^. The authors suggested that the differences in the pattern of activities from previous studies represented the demands of collaborative improvisation. They suggested this collaborative interaction involved distinct musical communication. Further they suggested that neural activity in the temporal-parietal junction as well as other cortical areas might have modified the demands on cortical processing of performance^1^ as seen previously^13,24^.

These foundational studies suggest intriguing insights into how distributed patterns of neural activity in the PFC can influence creative behaviors related to music, however it can be argued that it is difficult for an artist to perform truly creative and improvised music in the restricted environment of the fMRI scanner. In the limited space with loud scanning noise, subjects must adapt to the unfamiliar and uncomfortable surroundings while remaining extraordinarily still. The additional focus on these non-performance tasks may activate neural systems in opposition to those proposed for creative musical production and could actively compete for neural resources if the musicians are not effectively trained to perform in the MRI. A more naturalistic environment where musicians can use their personal instruments may allow subjects to behave similarly to their normal environment, leading to increased creativity. In this study, fNIRS was utilized as our group has reported numerous advantages over fMRI as a neuroimaging method in several domains^26,27^. Specifically, fNIRS is less sensitive to motion artifact than fMRI and allows subjects to behave more as they would in a naturalistic environment. The use of optical absorbance, in comparison to magnetic resonance, allows for investigations of how the brain responds during naturalistic interactions with instruments containing metal such as a guitar. While fNIRS does rely on the same slow hemodynamic responses as fMRI, the increased sampling rate associated with fNIRS may allow for determination of changes in neural responses with increased temporal granularity. Pressings model of improvisation argues for an ongoing balance of neural processing of perceptual feedback and error correction during improvisation^1,4^. This type of regulation could happen in executive control centers in the PFC and depending on the difficulty of a task cognitive load in the PFC can be modulated throughout the task depending on the performance. The cognitive load associated with the balance of these processing streams may be reflected in the time course of the responses and previous studies have shown this high sampling frequency can provide us with additional information regarding hemodynamic responses^28^. fNIRS with a sampling rate of 100 times that of fMRI allows us to investigate with greater temporal acuity these types of ongoing changes in responses within in a bout of activity and allows us to correlate them with changes in behavior.

In addition to exploring neural activity elicited in ecologically valid interactions of the musician with their instrument and background music, an additional goal of this study was to compare domain generalizability of the role of the DLPFC as found in previous studies^1,13,19–21,25^ to musical creativity in other genres of music. In this study we chose to investigate blues-rock due to its simplicity and the relative ease that even beginner guitar players are able to improvise within the rules of the genre^29,30^. Blues rock is one of the fundamental rock music (rock ‘n’ roll) and constructed by simple musical theory and often composed of ‘standard twelve-bar blues’ chord progression^30,31^. This simplicity and adaptability provide features that are suitable for developing experimental models of functional neuroimaging to explore the production of artistic creativity in musicians with a variety of skill levels.

Our goal was to determine brain areas in the PFC that respond to improvisation compared to simple formulaic play in musicians with a wide range of guitar skill. A blues rock background music (BGM) track was used to assist with tempo and musical interaction and to serve as audio information for the player to utilize for improvisation as well as to provide rules for formulaic sequence performance as has been used previously in other musical genres^13,19,25^. We hypothesized that activity in the DLPFC would show similar patterns of deactivation during improvised sequences compared to previous studies investigating piano and vocal improvisation^24^. Further we also hypothesized the simplified blues-rock genre would show similar results in DLFPC activity between all subjects regardless of skill level.

## Results

### Behavior

To determine any differences in amount of motor behavior which could influence neural responses we quantified the number of string picking events to Improv (improvise play) and Formulaic (play predetermined formulaic music scale) conditions in twenty subjects. The number of string picking (mean [standard deviation (SD)]) in each condition Improv and Formulaic was 115.29 [18.83] and 114.20 [29.73], respectively. A paired t-test revealed no significant differences in total number of picking of strings between tasks (p = 0.85).

### Event-triggered averages for task versus baseline

**Figure 1:**
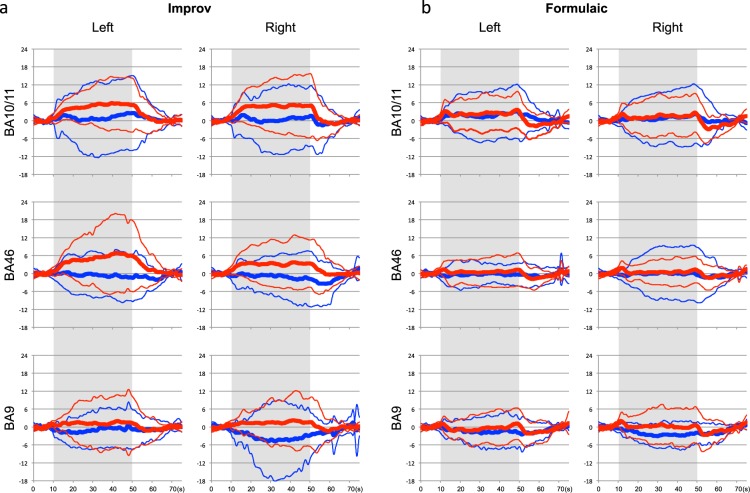
Averaged hemodynamic responses of three prefrontal areas (BA9, BA46, and BA10/11) in left and right hemispheres for Improv (**a**) and Formulaic (**b**) conditions. Time-courses of oxyHb and deoxyHb responses are shown in red and blue lines, respectively. Thick and thin traces represent the mean response amplitude and its 1SD ranges. Grey areas indicate the task duration.

Figure 1a shows plots of the averaged activation patterns for oxyHb and deoxyHb on the frontopolar cortex (FPC; Brodmann area [BA] 10/11) and the DLPFC (BA46 and BA9) in both hemispheres during the Improv task in 20 subjects. Significant activity versus baseline was observed for all tasks for both oxyHb and deoxyHb responses (p < 0.05), except for deoxyHb in the left BA10. Figure 1b shows the equivalent plots of the averaged activation patterns for oxyHb and deoxyHb in the FPC and the DLPFC in both hemispheres during the Formulaic task. Significant activity versus baseline averages were observed for all tasks for both oxyHb and deoxyHb responses (p < 0.05).

### Event-triggered average contrast of Improv > Formulaic tasks

**Figure 2:**
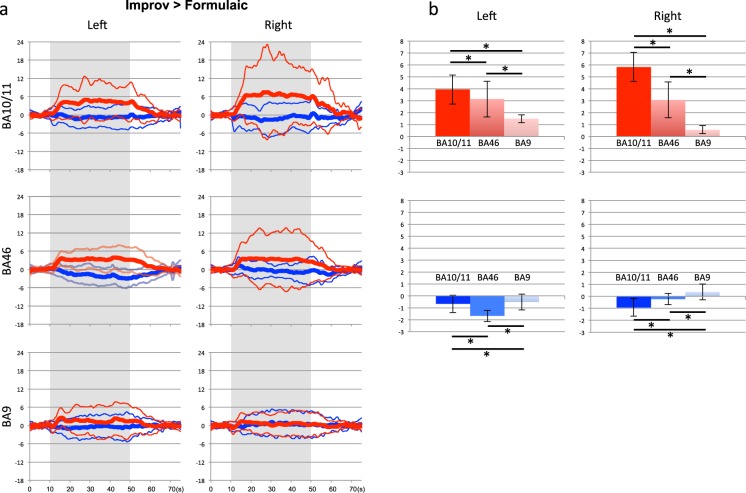
Hemodynamic response differences between Improv and Formulaic conditions in the prefrontal regions of interest (BA9, BA46, and BA10/11). (**a**) Averaged time-courses of hemodynamic responses by subtraction analysis from Improv to Formulaic of three prefrontal areas in the left and right hemispheres. Red and blue lines show oxyHb and deoxyHb responses, respectively. Thick and thin traces represent the mean response amplitude and its 1 SD ranges. Grey areas indicate the task duration. (**b**) Statistical comparisons of the mean oxyHb and deoxyHb amplitudes during task periods for the differential responses subtracted from Improv to Formulaic. Asterisk shows statistically significant differences between the regions (paired t-test with Bonferroni correction; p < 0.05). Average values are shown in red and blue bars, respectively. Each error bar shows +/− 1 SD.

Figure 2a demonstrates the event-triggered average plot (+/−SD) of the results of the contrast Improv > Formulaic tasks from the FPC and DLPFC for the left and right hemispheres. All contrasted responses show the task to be significantly greater than baseline (p < 0.05). Additionally, within each hemisphere we compared oxyHb and deoxyHb responses for each of the Brodmann areas to that of the other areas. Activity in each region was found to be significantly increased for both oxyHb and deoxyHb responses between all areas (p < 0.05) compared to baseline. Figure 2b shows the result of this comparison.

### Detailed temporal analysis of Improv versus formulaic responses

We compared the beginning, middle and end of each task between conditions to determine potential differences in cognitive processing associated with the ongoing task. To determine temporal differences in hemodynamic responses between Improv and Formulaic performances, we divided the responses into 10 s bins. We also discarded the first and last 5 s of the response for these comparisons to assure equal bins. Amplitudes of oxyHb and deoxyHb signals were compared between each bin within each BA and hemisphere of the PFC with a two-way, repeated measures ANOVA followed by Tukey’s multiple comparisons test with Bonferroni correction. For oxyHb, no significant interaction was found between time bin and condition (i.e., Improv and Formulaic) in any of the PFC areas. For deoxyHb, a significant interaction between time and condition (F(2, 38) = 3.366, p = 0.0451) was found in the right BA9 (BA9R), and (F(2, 38) = 6.519, p = 0.0037) left BA46 (BA46L). Post-hoc multiple comparisons showed significant differences for BA9R between Improv and Formulaic conditions in 25–35 s time bin (p = 0.0035). In BA46L post hoc comparisons found significant differences between bins 5–15 s vs. 25–35 s and 15–25 s vs. 25–35 s in the Formulaic condition. Additional significant differences between Improv and Formulaic conditions were found in 25–35 s time bin (p = 0.0012).

### Correlation between the questionnaire and hemodynamic signals

**Figure 3:**
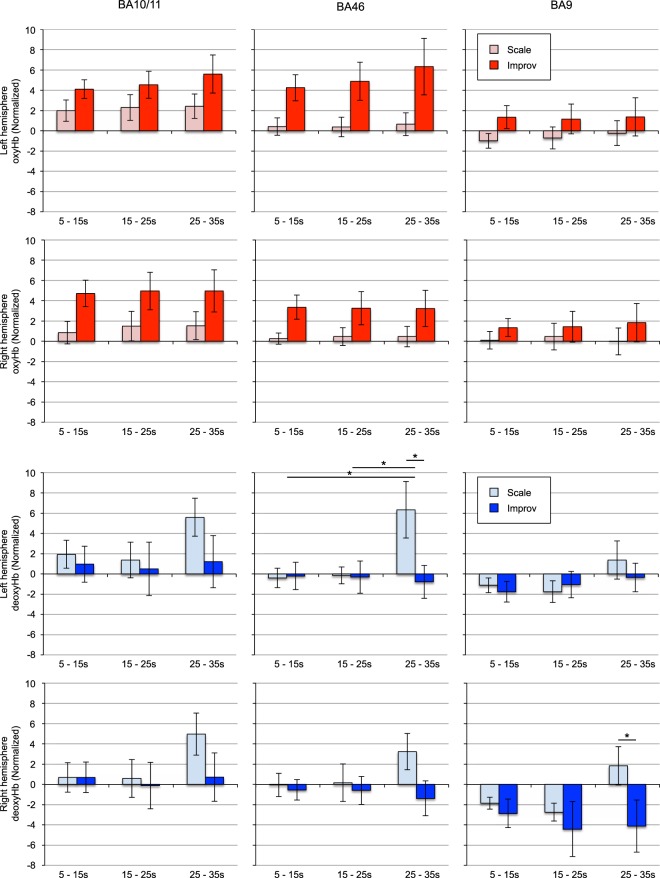
Comparison between Formulaic and Improv for oxyHb (red bar) and deoxyHb signals (blue bar) averaged every 10 s in the three prefrontal areas (BA10/11, BA46, and BA9). Time legends indicate the time elapsed from the task onset. Each value shows mean +/− SEM (standard error of the mean). Asterisk indicate statistically significant difference between time bins (p < 0.05, corrected).

**Figure 4:**
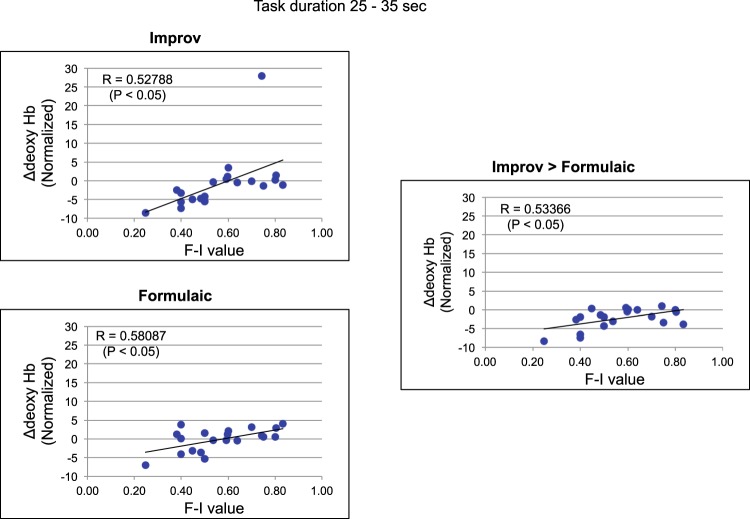
Correlation between F-I value and deoxyHb responses of the left BA46 averaged during 25–35 s for all participants.

Finally, we attempted to determine how skill, history, age, or subjective feeling influenced the guitar task and neural activity. We determined correlation between the subjective creative index (F-I value), difficulty, history, age, and practice versus hemodynamic contrasts, which indicated significant differences between Improv and Formulaic compared to binned time courses (Fig. 3). No correlations were found for oxyHb or deoxyHb signals with respect to difficulty, history, age, or practice for any time bin. We found a high correlation for deoxyHb responses in the BA46L versus F-I value in the Improv > Formulaic, Improv > Baseline, and Formulaic > Baseline tasks (p < 0.05; Table 1 and Fig. 4).

**Table 1:** Correlations between three PFC areas (i.e., BA10/11, BA46, and BA9) and scores from post-experimental questionnaire.

Left Hemisphere																
oxyHb	PFC area	r value														
bin		F-I value			Difficulty			History			Practice			Age		
		Imp	Form	Subtract	Imp	Form	Subtract	Imp	Form	Subtract	Imp	Form	Subtract	Imp	Form	Subtract
5–15s	BA10/11	0.152	0.084	0.049	0.058	0.018	0.015	0.228	0.051	0.137	0.356	0.123	0.132	0.232	0.150	0.138
	BA46	0.229	0.161	0.369	0.088	0.224	0.257	0.226	0.215	0.149	0.163	0.274	0.314	0.162	0.221	0.107
	BA9	0.217	0.047	0.309	0.057	0.064	0.003	0.196	0.056	0.371	0.290	0.176	0.304	0.162	0.055	0.326
15–25s	BA10/11	0.161	0.053	0.034	0.069	0.082	0.023	0.179	0.186	0.203	0.241	0.136	0.046	0.190	0.302	0.192
	BA46	0.235	0.182	0.439	0.133	0.005	0.290	0.184	0.255	0.008	0.094	0.336	0.089	0.131	0.322	0.043
	BA9	0.265	0.055	0.375	0.066	0.177	0.016	0.095	0.204	0.219	0.152	0.200	0.113	0.064	0.246	0.170
25–35s	BA10/11	0.084	0.137	0.013	0.114	0.224	0.054	0.139	0.256	0.202	0.171	0.257	0.023	0.141	0.379	0.182
	BA46	0.196	0.150	0.212	0.205	0.075	0.092	0.179	0.144	0.100	0.039	0.323	0.045	0.122	0.223	0.071
	BA9	0.182	0.154	0.279	0.075	0.193	0.015	0.102	0.139	0.236	0.149	0.226	0.078	0.078	0.207	0.201
**Right Hemisphere**																
**oxyHb**	**PFC area**	**r value**														
**bin**		**F-I value**			**Difficulty**			**History**			**Practice**			**Age**		
		**Imp**	**Form**	**Subtract**	**Imp**	**Form**	**Subtract**	**Imp**	**Form**	**Subtract**	**Imp**	**Form**	**Subtract**	**Imp**	**Form**	**Subtract**
5–15s	BA10/11	0.109	0.030	0.285	0.139	0.137	0.398	0.282	0.369	0.065	0.210	0.318	0.064	0.219	0.260	0.095
	BA46	0.259	0.346	0.446	0.255	0.326	0.031	0.325	0.357	0.070	0.170	0.265	0.080	0.294	0.327	0.119
	BA9	0.018	0.102	0.323	0.109	0.062	0.035	0.042	0.131	0.320	0.336	0.044	0.491	0.001	0.102	0.326
15–25s	BA10/11	0.015	0.054	0.189	0.101	0.030	0.354	0.361	0.183	0.143	0.236	0.286	0.016	0.287	0.066	0.167
	BA46	0.209	0.363	0.394	0.198	0.069	0.003	0.353	0.055	0.160	0.228	0.076	0.135	0.304	0.067	0.209
	BA9	0.124	0.118	0.261	0.003	0.230	0.053	0.117	0.003	0.236	0.162	0.052	0.461	0.076	0.039	0.243
25–35s	BA10/11	0.065	0.247	0.140	0.083	0.065	0.356	0.351	0.245	0.179	0.186	0.229	0.053	0.269	0.108	0.189
	BA46	0.138	0.143	0.292	0.177	0.057	0.065	0.354	0.112	0.109	0.215	0.047	0.123	0.296	0.019	0.159
	BA9	0.227	0.079	0.118	0.038	0.170	0.012	0.137	0.165	0.203	0.129	0.068	0.425	0.099	0.073	0.200
**Left Hemisphere**																
**deoxyHb**	**PFC area**	**r value**														
**bin**		**F-I value**			**Difficulty**			**History**			**Practice**			**Age**		
		**Imp**	**Form**	**Subtract**	**Imp**	**Form**	**Subtract**	**Imp**	**Form**	**Subtract**	**Imp**	**Form**	**Subtract**	**Imp**	**Form**	**Subtract**
5–15s	BA10/11	0.439	0.454	0.327	0.005	0.019	0.034	0.076	0.009	0.336	0.335	0.433	0.195	0.000	0.072	0.274
	BA46	0.453	0.203	0.553	0.085	0.318	0.226	0.174	0.240	0.304	0.111	0.336	0.275	0.068	0.351	0.257
	BA9	0.443	0.301	0.416	0.182	0.115	0.131	0.354	0.040	0.506	0.117	0.491	0.299	0.235	0.069	0.485
15–25s	BA10/11	0.411	0.463	0.353	0.004	0.004	0.107	0.064	0.014	0.158	0.326	0.428	0.382	0.003	0.086	0.126
	BA46	0.599	0.287	0.747	0.100	0.285	0.293	0.163	0.270	0.185	0.250	0.277	0.361	0.079	0.362	0.163
	BA9	0.545	0.409	0.408	0.118	0.079	0.081	0.387	0.081	0.541	0.033	0.518	0.377	0.328	0.125	0.540
25–35s	BA10/11	0.378	0.490	0.120	0.100	0.059	0.438	0.063	0.038	0.060	0.358	0.455	0.469	0.016	0.062	0.014
	BA46	0.528	0.581	0.534	0.041	0.184	0.140	0.227	0.023	0.138	0.222	0.005	0.246	0.135	0.049	0.084
	BA9	0.522	0.484	0.255	0.171	0.055	0.189	0.431	0.045	0.539	0.047	0.471	0.280	0.417	0.069	0.558
**Right Hemisphere**																
**deoxyHb**	**PFC area**	**r value**														
**bin**		**F-I value**			**Difficulty**			**History**			**Practice**			**Age**		
		**Imp**	**Form**	**Subtract**	**Imp**	**Form**	**Subtract**	**Imp**	**Form**	**Subtract**	**Imp**	**Form**	**Subtract**	**Imp**	**Form**	**Subtract**
5–15s	BA10/11	0.487	0.354	0.045	0.018	0.144	0.017	0.100	0.163	0.274	0.245	0.170	0.034	0.005	0.051	0.276
	BA46	0.368	0.076	0.308	0.297	0.221	0.203	0.240	0.061	0.365	0.629	0.619	0.264	0.234	0.056	0.368
	BA9	0.135	0.148	0.230	0.172	0.242	0.284	0.300	0.123	0.524	0.058	0.117	0.367	0.256	0.011	0.590
15–25s	BA10/11	0.503	0.273	0.346	0.057	0.135	0.061	0.035	0.232	0.040	0.389	0.104	0.341	0.105	0.115	0.054
	BA46	0.367	0.002	0.309	0.221	0.160	0.220	0.138	0.025	0.254	0.642	0.656	0.170	0.165	0.005	0.254
	BA9	0.120	0.118	0.230	0.169	0.138	0.325	0.187	0.015	0.470	0.055	0.095	0.320	0.158	0.092	0.534
25–35s	BA10/11	0.446	0.284	0.206	0.104	0.141	0.060	0.021	0.303	0.016	0.380	0.160	0.225	0.074	0.199	0.051
	BA46	0.247	0.017	0.166	0.114	0.152	0.094	0.184	0.074	0.181	0.614	0.722	0.199	0.245	0.103	0.282
	BA9	0.100	0.199	0.062	0.206	0.060	0.281	0.183	0.136	0.288	0.046	0.033	0.202	0.161	0.051	0.361

Only bin 25–35 s for BA46L (deoxyHb) versus F-I value shows high correlations (r > 0.5) in all three conditions, i.e., Improv > Formulaic (Subtract), Improv > Baseline (Imp), and Formulaic > Baseline (Form) tasks.

## Discussion

To our knowledge, this is the first study to explore the brain activity underlying creativity in the production of rock music played on a guitar. While it is difficult to argue the obvious benefit of studying rock music compared to other genres, many individuals have reported similar creative mental imagery associated with the rock music genre including terms to describe rock music as ‘freedom’, ‘powerful’, ‘violent’, ‘destructive‘, ‘loud’, ‘brave’, ‘vital’, and ‘live’^29–33^. This emotional imagery suggests rock music may have the potential to generate specific elements of creativity when compared to other genres of music for both the player and listener. The specific sub-genre of rock music, blues rock, has a simple musical chord construction and can be readily and quickly learned by guitar players at almost any skill level if the player knows how to play the pentatonic scale. While the basic idea that the neural mechanisms associated with spontaneous musical creativity should be similar across artistic activities, a simplified task and genre allows for a more reductionistic approach to studying creativity and should provide similar results across multiple types of musicians with a wide range of skills.

In the present study, we utilized fNIRS to assess hemodynamic responses in the PFC during improvised compared to formulaic play on an electric guitar. To control for behavior across the two tasks, we developed two conditions to ensure that improvised play (Improv) included numerous elements of musical creativity compared to formulaic play (Formulaic) and that the Formulaic condition included minor elements of improvisation as described in the Methods to assure the sequence was not too simplistic. Aside from these improvised elements, other structures of guitar interaction were controlled for to ensure changes in hemodynamic signals between conditions would be specific to spontaneous creativity in comparison with other motor or artistic activities of guitar play. Tempo and picking timing were influenced by the blues rock BGM track. An analysis of behavior regarding the number of picks indicated no difference in the amount of picking between Improv and Formulaic conditions. All guitarists were able to perform the tasks regardless of skill.

Our goal for the two tasks was to specifically isolate and amplify elements of spontaneous creativity occurring in a short sequence of play that could be repeated several times in order to increase the signal to noise ratio of the hemodynamic response. To do this, players in the improvised task blocks were first required to start an impromptu performance and as the task progressed, they needed to recall and concatenate previously utilized chunks of melodic phrases to match the BGM track and their previous play^1,13^. This process is constantly required to overwrite and renew chunks of melodic phrases during Improv task blocks (40 s). This constant updating of working memory with new sequences of motor behaviors can be compared Pressing’s balance of sensory and perceptual encoding, motor control, performance monitoring, and memory retrieval^4,5^. Execution of the Formulaic task did not require this type of repeated melodic phrasing and chunking to occur, with unique motoric chunking events only happening sporadically.

Our investigation of cortical responses focused on the PFC, which has been previously reported as a higher-order neural network and is shown to be involved in musical and other types of creativity^10,13,16–18^. The PFC is functionally subdivided on the underlying cytoarchitecture defined by BA; each area may contribute independent elements of the creative process^13,34^. Recent reports investigating brain function in the PFC related to general aspects of creativity have shown a unique neural pattern that is not typical of traditional cognitive tasks^13–15^. Specifically, it has been suggested that during creative musical performances, widespread deactivation is seen in the lateral PFC, including the DLPFC, while significant activation of the FPC is observed^13,24^. In the present study, we attempted to replicate this pattern of activity in improvised guitar play by investigating specific hemodynamic differences in the FPC (BA10/11), the DLPFC (BA46 and BA9).

Our first goal was to perform an investigation of the recorded data to determine general aspects of the hemodynamic response in the Improv > Formulaic contrast. We first event-triggered all responses and determined the activity in oxyHb to be greater than baseline for the three areas. The results indicated that these three areas showed activity greater than others (p < 0.05), which only partially supports the results of previous studies, but does replicate that of others^1^. Results for this contrast in activated areas also displayed specific cortical activity patterns showing inversely correlated oxyHb and deoxyHb responses consistent with hemodynamic signals associated with underlying neural activity^35^. Although BA10/11 was consistent with previous reports^13^ of musical creativity showing significant activity in the Improv > Formulaic contrast, BA9 and BA46 responses were less consistent and showed increased variability across subjects. We also compared responses across the left and right PFC for significant differences between the three prefrontal areas. Results indicated all three bilateral sub regions showed significant differences from each other for both oxyHb and deoxyHb signals (p < 0.05) suggesting different roles for each area in improvised guitar play, but not necessarily specific deactivation of the areas as found previously.

The increased pattern of activity in BA10 during the creative process is consistent with multiple reports on creativity and is suggested to be related to top-down regulation^22^. It may also play a crucial role in “predictive coding”^36,37^, regulation of “prospective memory”^38^ and artistic creativeness^13,15^. The role of BA10 may also function as a memory buffer that holds intention of future behavior until the appropriate timing for execution based on environmental feedback. It has also been shown through structural imaging data that musically creative people have greater cortical surface area or volume in emotion-related regions, including the FPC^27^. The guitar play used in this study required this type of prospective memory with respect to integration of motor intention and tempo (i.e., the players had to prepare to pick strings with their right hand, hold the strings of the fingerboard with their left-hand fingers, and listen to the BGM track). The FPC has also been suggested to play a role in supramodal regulation, especially in cases in which outcomes of two or more separate cognitive operations need to be integrated to achieve a higher behavioral goal^39^ or in coordination of multiple cognitive processes^22^. The pattern of using both hands to perform separate tasks on the guitar supports this type of integration of multiple processes. The activity shown in BA10/11 also specifically supports the findings of Limb and Braun for jazz pianists within the FPC as well as Liu *et al*. for vocalists, where it has been suggested that increased activity of the FPC during improvisation plays a role in internally motivated and self-generated behaviors^13^.

Analysis of group results of BA46 and BA9 did not replicate previous findings on jazz piano or vocal creativity but does fit other models of creativity proposed for more general tasks such as drawing and musical tasks where more than one musician is involved. BA46 showed positive activity for the Improv > Formulaic task. BA9 responses were reduced during improvisational play for some subjects and negative for others; however, a consistent deactivation of the dorsolateral and dorsomedial prefrontal cortices was not found in the guitar task such as in the jazz piano improvisation^13^. While this finding is not consistent with the results of Limb and Braun^13^ and Liu *et al*.^24^, the significant differences in activity found between each area do suggest improvised creativity may indeed employ independent processing centers within the PFC. It is also possible that experience may have played a role in this difference in deactivation^20^ as has been found in other studies on pianists^20,21^. It is possible that the previous report by Limb and Braun^13^ focused on six professional pianists, which may represent a case of sophisticated players developing a neural circuit that is specific to their ability and not representative of a more general creative process. Even in the Pinho *et al*. study, the recruited pianists still had a university degree in piano performance or were students in musical schools with training in classical and/or jazz piano playing. On the other hand, in our study greater variation of guitar skill as well as the variability in neural activity in the DLPFC from the participants with a guitar playing history ranging from 3 to 43 years may represent a more generalizable creativity. Finally, it is also possible that the environment of the fMRI scanner played a role in the production of the activity pattern seen previously. This could be due to competing cognitive processes that allow pianists to play laying down without seeing the keyboard and with loud MRI noises.

An alternative explanation for the variation in responses from the DLFPC may be explained by differences in ongoing cognitive processing involved in the balancing of the melodic phrasing and chunking between subjects. The F-I value behavioral measure suggested that subjects did not always feel they improvised well and that often they felt that even when they were attempting to be improvisational the sequence was derived entirely from memory and was purely formulaic. We hypothesized that if this the subjects had increased neural activities to improvise it would come at the end of the 40 second sequence rather than at the beginning due to increases in cognitive load associated with memory recall. To further investigate the source of variability in responses from guitarists, we attempted to take advantage of the high sample rate of fNIRS to inspect the temporal detail of the hemodynamic response curves during task blocks. To leverage this sampling rate, we divided the average responses into 10 s bins to compare the specifics of the temporal nature of the beginning, middle and end of the hemodynamic signals (Fig. 3). Compared to BA10/11 and 46, the overall response of BA9 was reduced within the 40 s block in oxyHb, but when divided into smaller temporal bins, in the latter half of task durations deoxyHb in BA9L, BA9R and BA46L showed significant differences between conditions (i.e., Improv and Formulaic tasks) and between bins (i.e., 15–25 s vs. 25–35 s and 5–15 s vs. 25–35 s) in the block. The relationship between DLPFC (BA9 and BA46) and FPC (BA10/11), as well as other areas in the PFC, may function as a central processor to undertake self-monitoring and conscious volitional control of ongoing performance^13,40^. This suggests that the DLPFC has a role in regulation of artistic activity and exploration^19^ and may work directly to control self-generated behaviors related to the frontal pole and the default mode network. To be observed the relationship between the DLPFC and the FPC in the latter portion of the task (bin 25–35 s) in musical terms can be referred to as the time taken to lift the player to mental exaltation or where they reach their peak creativity. It is possible that this balance of internal motivation and external cueing may be more affected in the second half of the block than the first half and may be related to control of simultaneous execution of several processes in real-time, including sensory and perceptual encoding, motor control, performance monitoring, and memory retrieval, among others as suggested by Pressing^4,5^.

To determine a source for variability seen in responses in Figs 1 and 2 we explored correlation with our self-report measures. A significant correlation of subjective feeling of creativity with deoxyHb in BA46L suggests that when the subjects subjectively felt that they improvised well, the neural activity in BA46L was reduced, regardless of skill. Alternatively, when subjects subjectively felt they performed formulaic (non-creatively), the activity was increased. This correlation is true even for the task versus rest correlations when comparing F-I value versus the BA46L. (Improv > Formulaic (r = 0.528); Improv > Baseline (r = 0.581); and Formulaic > Baseline (r = 0.534)). These correlational relationships suggest that subjective feelings of creativity during improvisation may be a key factor in regulation or influence by the DLPFC as opposed to age, skill, or number of hours of practice. We suggest that when even beginner players feel that they improvise well, it may actually reflect the same deactivation of the DLPFC as found previously by Limb and Braun^13^ and Liu *et al*.^24^. This deactivation in the DLPFC may involve ongoing analysis of one’s own performance. The ability to quickly reach “mental exaltation” or to release focus on external cues may be important in this deactivation of the DLPFC. Pinho *et al*. suggested brain activation during improvisation compared to rest indicated a correlation in DLPFC activity with self-reported lifetime improvisation hours^1,20^. They also showed improvisational expertise was negatively correlated with activation in a right-lateralized network of brain regions, including the DLPFC, IFG, anterior insula, and angular gyrus^20^. In addition, they also indicated experts showed greater functional connectivity between the DFLPC and premotor cortex during improvisation (i.e., bilateral PMD and pre-SMA). They interpreted the relative deactivation of executive control networks in experts may correspond to an overall automation of domain-specific cognitive processes and increased functional connectivity between frontal regions may translate to more efficient access to pre-learned motor patterns and generative strategies stored in long-term memory. This finding is quite similar to studies investigating video gaming by experts with cumulative suppression of the FPC^22^. This automation of domain-specific cognitive processes will be essential for highly skilled play, but not be necessarily to generate creativity in general.

As alternative perspective reported by Donnay *et al*. found increased activation of the DLPFC during collaborative improvisation in contrast to individual improvisation^1,13,25^. The authors noted that the nature of collaborative improvisation appears to be different from solo improvisation, and suggested that collaborative improvisation involves a distinct type of musical communication that requires greater demands on performance monitoring and perhaps integration of social cues as an additional cognitive load. Again, however, this difference in demand on performance monitoring can be explained in terms of the differences in subjective feelings related to performance between collaborative and solo improvisation. We suggest that the nature of improvisation can be influenced by subjective creative feeling in general compared to solo vs. collaborative or lifetime improvisation hours (practice). For example, children can draw very imaginative pictures using pure creativeness even though they have never had specific training regards to art. It can be argued that this is a pure form of improvisation that cannot be explored in trained experts.

Further study is required to explore the relationship between specific distributed patterns of neural activity in the PFC and improvised musical performances. In the present study, we have found support for aspects of the framework proposed by Pressing regarding improvisation^4,5^. We have also found similarities in neural responses when comparing improvised behavior in blues rock guitar to formulaic sequences as previously found for piano and vocals^13,24^. We have also shown how studying a wider range of skills during performances may provide additional insight to the complex nature of how the circuits in the PFC and default mode network are modulated. Our results add to this growing field in that we have proposed a method to study a wider range of musicians using a simplified and naturalistic task. Variability associated with performance does make the interpretation of the results more difficult, but also suggests what specific roles each processing center in the PFC may perform with more granularity. Our goal is to expand on this exploratory study and collect larger samples that can be further divided into musical skills, as well as explore other genres of music. However, here we could suggest that rock music or other more popular artistic creative works, including pop music, can be useful to explore brain circuits related to creativity. We argue that it is important when studying creativity to not only investigate limited special populations such as professional musicians with years of expertise, but also to explore these same circuits in ordinary people using as task constructed by simple popular musical theory like rock ‘n’ roll.

This study has a number of limitations that need to be addressed. First, while we were able to recruit 20 subjects, the sample size is insufficient to make general inferences regarding the creative process. Recruitment at a local guitar club also tended to recruit only male subjects; therefore, we also want to balance the ratio of males to females to assure the pattern of activity is not exclusive to males. Second, because we had only the questionnaire with five categories in this study, there may be a possibility of a superior correlation factor for spontaneous creativity. In the future we will ask for additional demographic information to attempt to address these issues including how much time musicians practice improvisation. Additionally, we did not employ a systemic artifact removal, as the Hitachi 4000 fNIRS topography system does not have the ability to create short optode channels. In the future we plan to integrate a spatial filter as suggested by Zhang *et al*.^41,42^, but since we focused on contrasted tasks with similar behaviors (number of guitar picks was nearly identical across subjects) we feel any artifact would have been equal in both tasks^43^. It is possible that the oxyHb signals may contain increased blood pressure artifacts associated with the act of focused improvisation on the guitar and may have increased variability in oxyHb signals. However since we found characteristic counter correlation between oxyHb and deoxyHb signals we feel the results presented here represent spatial patterns of neural processing.

## Methods

### Subjects

Twenty healthy, amateur or professional musicians (aged 19–63 years; mean: 34.5; SD: +/− 13.261; 100% male; 3–43 years of practice) volunteered to participate in the study. The skill levels were wide-ranging, but each subject reported the ability to play blues rock with improvisation. Subjects were recruited from the Light Music Club at Dokkyo Medical University. Seventeen participants were right-handed, two left-handed, and one was ambidextrous. While handedness was not uniform, all musicians did report as playing standard right-handed style guitar (i.e., picking strings with a plastic pick with their right hand and holding strings on the fingerboard with their left hand). Handedness in each subject was determined by a post-experimental questionnaire^44^. No subject reported any history of neurologic or psychiatric disorders. Informed consent was obtained for all subjects and the research protocol was approved by the Institutional Review Board at Dokkyo Medical University (approval No. 26002).

### fNIRS measurement and channel localization

**Figure 5:**
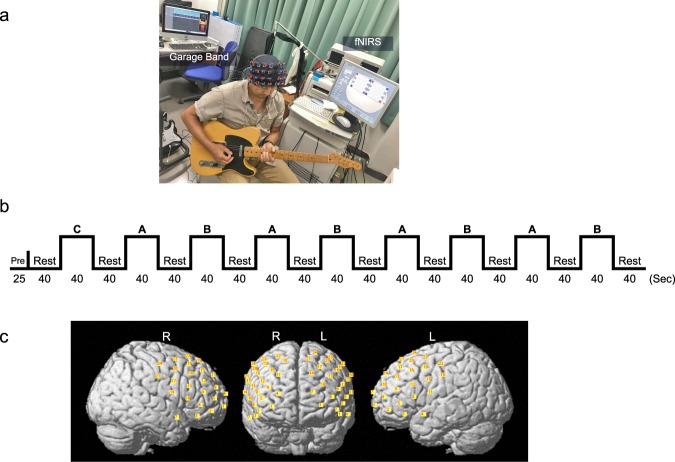
Experimental settings. (**a**) A naturalistic environment of the experiment. Participants play a real guitar while their brain activity is scanned with fNIRS. (**b**) Task Paradigm. A: Improv; B: Formulaic; C: Sham (Same as B, but not used in analysis). (**c**) An optical probe arrangement of a representative participant. Probe array positioned over the left and right prefrontal lobes including BA10/11, BA46, and BA9. Each red number shows optode channels.

Recordings were obtained using a 48-channel ETG-4000 fNIRS topography system (Hitachi Medical Co.) arranged into double 4 × 4 optical probe arrays (i.e., one optical probe array has 24-channels) on the fronto-lateral aspects of the subject’s head, as described in previous studies^22^. The arrays were mounted on an elastic optode cap and positioned over the left and right PFC, including BA10/11, BA46, and BA9 (Fig. 5c). Inter-optode distance was 3 cm for each source detector pair and recordings were sampled at 10 Hz. The anatomical locations of all optodes were recorded in relation to standard head landmarks including inion, nasion, top center, and left/right tragi using a Patriot 3D Digitizer (Polhemus) and linear transform techniques as previously described^27^.

The coordinates of the head landmarks and probe positions were used to estimate the position of each channel in the Montreal Neurological Institute standard brain space^45,46^. Two regions of interest (ROI), the FPC and DLPFC, reported to be active and inactive in creative music processing were selected for this study^13,24^. Channels for the ROI were chosen and analyzed based on 3D digitizer information. For the channel-wise approach, only channels were analyzed for oxyHb and deoxyHb that showed a registration probability of 80% or more in the BA10/11, BA46, and BA9 in left and right hemispheres according to the output of the registration process. BA10/11, BA46, and BA9 correspond to the FPC, the LPFC, and DPFC, respectively. The channel with the highest percentage was used when more than one channel was found with greater than 80% probability.

### Task conditions and paradigm

The goal of the study was to determine neural responses during a unique and ecologically valid blues-rock guitar task comparing improvised to formulaic sequences. We created a modified paradigm, similar to that used in previous studies^1,13^, in which subjects played blues rock music in a jam session style on an electric guitar (Telecaster, American Vintage ‘52, Fender USA) while neural activities were continuously measured by fNIRS. Subjects performed the guitar task while listening to a BGM track (through headphones) that was made up of a standard twelve-bar blues chord progression that was meant to guide their play and help control the number of individual guitar picks. This BGM track was made using Garage Band (Apple Inc.) and was composed of 40 s of slow blues rock at 108 beats per minute in the key of E minor (One loop is from roughly 10 to 50 s in (Supplemental Auditory Example 1). This blues rock loop was fit to a block design consisting of 40 s of activity alternating with 40 s of rest (Fig. 5b).

Two versions of the blues rock guitar task were developed that comprised a modified paradigm, similar to the jazz piano task of Limb and Braun^13^ and the rap vocal task studied by Liu *et al*.^24^. In an attempt to generalize findings in a format similar to the review article by Beaty, 2015^1^, we compared improvised play to a more formulaic pattern easily playable by memory alone. In the present study, subjects performed two tasks. The first task was termed Formulaic (play the predetermined and well-practiced formulaic E minor pentatonic scale including the blue notes), and the second was termed Improv (improvise with the E minor pentatonic scale including the blue notes). The tasks were performed in a block design, repeating the Formulaic task five times and the Improv task four times. Each task was alternated and rest periods (listening to the BGM track only with no physical guitar play) for 40 s were interleaved between each task (Fig. 5b). The first run of the Formulaic task was discarded for data analysis because it was used for participants to adapt to the experimental environment and to avoid cortical blood flow changes that may introduce systemic artifact due to increases in blood pressure or respiration associated with the task^41–43^. To control for the sensorimotor circuits in both tasks, participants were asked to restrict their finger and hand movements between frets 12–15 in both the Formulaic and Improv task conditions. To further control for behavior between the two tasks, subjects were also required to demonstrate bending and vibrato (as in improvised play) at regular intervals during the Formulaic condition, while they repeated the E minor pentatonic scale sequence (including blues notes used in the Improv task) in ascending and descending order on the beat with the BGM track during the task sequence (The example of the Formulaic condition is from about 10 to 50 s in (Supplemental Auditory Example 2). During Improv, subjects were asked to play and improvise a melody at their discretion but were required to adhere to the beat of BGM track (The example of the Improv condition is from about 10 to 50 s in (Supplemental Auditory Example 3). Additionally, they were asked to maintain approximately the same number of picked notes between the Formulaic and Improv conditions. The number of picked strings during the Formulaic and Improv conditions for each subject was quantified to investigate any significant differences between task conditions.

### Data analysis

To compare conditions, we measured event-triggered averaged responses between tasks for oxy and deoxyHb signals. Both signals were contrasted against baseline to determine response to task and counter-correlation between oxy and deoxyHb signals was assumed to indicate localized neural processing. Additionally, a contrast Improv > Formulaic was determined and contrasted against rest to determine responses specific to the Improv condition. Changes in oxyHb and deoxyHb responses were averaged over four trials (for each Formulaic and Improv condition) using the built-in integration function of the fNIRS system (ETG-4000 V1.932; Pre: 10.0 s, Recovery: 15.0 s, Post: 10.0 s). A modified Beer–Lambert approach^47^ was used to calculate hemodynamic signals reflecting oxyHb and deoxyHb concentration changes in an arbitrary unit (mM mm). Signals from individual channels were low-pass filtered (0.05 Hz) and averaged. A linear regression by the least square method was performed based on oxyHb and deoxyHb responses between pre- and post-periods to serve as a baseline correction. The amplitude of the hemodynamic signal was further normalized by dividing oxyHb and deoxyHb values by the standard deviation of those during the 10 s before task onset. These baseline-corrected and normalized oxyHb and deoxyHb responses were used for further analysis^22,48^.

Processed oxyHb and deoxyHb signals for the task conditions were averaged across subjects for group analysis. The averaged oxyHb and deoxyHb signals were used for contrasting hemodynamic signals during the Formulaic condition from during the Improv condition as described above..

Due to the high sampling rate of fNIRS we also investigated detailed temporal responses related to the tasks. To do this, we subdivided responses into 10 second sections or bins to determine interaction between style of play (i.e. improvised vs formulaic) to beginning, middle and latter portions of the musical sequences independently using repeated measures ANOVA. The initial and last 5 s of each block were dropped to ensure equal distribution of duration to each bin (i.e., from 5–15 s, 15–25 s, and 25–35 s). A post hoc comparison with Bonferroni’s multiple comparison correction was performed between Improv to Formulaic play condition versus time bin to determine statistical differences among the fNIRS data between conditions. A corrected P value of 0.05 used for the level of statistical significance.

### Correlation of neural recordings with behavior

To explore correlational relationships between the three prefrontal areas (i.e., BA10/11, FPC; BA46, LPFC; and BA9, DPFC) and improvised or formulaic guitar play behavior, each participant was asked to answer a post-experimental questionnaire immediately after the fNIRS scan. This questionnaire included two Visual Analogue Scales and three questions regarding experience/historical information. Q1 queried the subjective strength of creativity during improvisation; it was represented by the subjective degree of formulaic-improvised feeling during the Improv task (F-I value). For example, if the subject felt they were able to perform well-improvised play during the session including high creativity, the subject marked a high value using a percentage representation. If the subject felt they did not perform using creativity, (i.e., similar to formulaic play), a low percentage was reported. Q2 was regarding the subjective feeling of task difficulty (Difficulty). If the subject felt the task was difficult to perform with improvisation during the Improv task, they reported a high value using a percentage representation, and inversely if they felt the task was easy. Q3 queried the age of the subject (Age) and Q4, the number of years the participants had been playing guitar for (History). Finally, Q5 was regarding the number of hours of daily guitar practice (Practice). Each value obtained was used to determine a correlation coefficient with the averaged amplitude of hemodynamic signals of the FPC and DLPFC during each task period (Improv or Formulaic). Using the Pearson product-moment correlation coefficient, an r value of 0.5 or more was defined as a high correlation between the questionnaire content and the averaged amplitude of hemodynamic signal in each bin (p < 0.05).

## Supplementary information

supplemental auditory example 1

supplemental auditory example 2

supplemental auditory example 3
